# Circular RNAs and mammalian follicular development: current insights and future prospects—an updated review

**DOI:** 10.1038/s41420-025-02661-z

**Published:** 2025-10-07

**Authors:** Huifeng Li, Tianle He, Dengjun Ma, Huanhuan Gong, Zhenguo Yang

**Affiliations:** Laboratory for Bio-feed and Molecular Nutrition, College of Animal Science and Technology, Southwest University, Chongqing, China

**Keywords:** Urogenital reproductive disorders

## Abstract

Circular RNAs (circRNAs), a newly recognized category of non-coding RNA, have recently become a central point of interest in biological research. The ovaries are critical reproductive organs in female mammals, profoundly influencing fertility through their effects on endocrine functions and follicular cycle activities. Follicle development, as the fundamental functional component of the ovaries, is elaborately regulated by granulosa cells, oocytes, and endocrine signals. Recent research has progressively underscored the critical role of circRNAs in regulating follicular development and maturation in mammalian species. This review comprehensively examines the formation, molecular characteristics, and biological significance of circRNAs during mammalian follicular development, with a specific focus on their regulatory mechanisms and functional patterns in this process. We propose that future research should continue to explore the specific mechanisms by which circRNAs influence follicular development in mammals, including their interactions with other non-coding RNAs, the mechanisms of their interaction with the follicular microenvironment, and the alterations in follicular environments under pathological conditions, including polycystic ovary syndrome (PCOS) and primary ovarian insufficiency (POI). Furthermore, we analyze the potential contributions of circRNAs in follicular development in view of advances in high-throughput sequencing technologies and gene editing tools, aiming to deepen our understanding of the biological significance of circRNAs in this context. In summary, this review elucidates the specific mechanisms and critical roles of circRNAs in follicular development in female mammals, potentially providing new therapeutic targets and strategies for future reproductive medicine and fertility treatments.

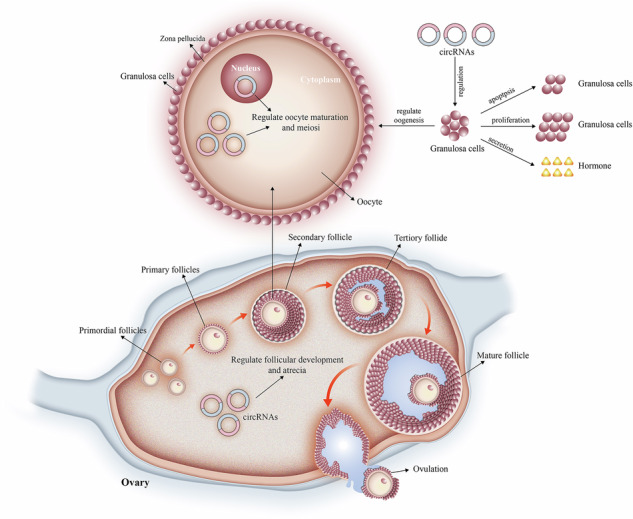

## Facts

- CircRNAs play a complex and crucial role in the development of mammalian follicles.
- Most existing studies are based on static follicle samples and lack investigation into the dynamic changes of circRNAs throughout the ovulation cycle.
- Sequencing and gene editing technologies are expected to advance the regulation of healthy follicular development by circRNAs.

## Open Questions

- Can a single circRNA regulate follicular development by targeting multiple miRNAs to initiate cascade reactions?
- Are the aberrant expression patterns of circRNAs observed in pathological conditions such as PCOS and POI a primary cause of disease, or do they represent a compensatory response?
- How do circRNAs regulate intercellular communication between granulosa cells and oocytes within the follicle, particularly through delivery via extracellular vesicles such as exosomes?

## Introduction

As reviewed, follicular development is a key process in ovarian function, with direct implications for female reproductive health and fertility [1]. Breeds (or individuals) of pigs with high fertility have a higher number of mature follicles in their ovaries compared to those with low fertility [2]. Follicular development involves the maturation of oocytes, and the precise regulation of granulosa cells (GCs), follicular fluid, and other microenvironmental factors. During this process, the interaction between GCs and oocytes is pivotal in the controlling follicle proliferation, differentiation, hormone synthesis, and eventual maturation [3, 4]. The healthy development of follicles not only determines egg quality, but also profoundly affects reproductive function and hormone balance in the body [5, 6]. Therefore, studying the developmental mechanisms and functions of follicles is important for addressing female infertility, optimizing livestock reproduction, and promoting human reproductive health.

Recently, as reviewed by scholars, circular RNAs (circRNAs), a noval class of non-coding RNA molecules, have attracted increasing attention in biological research. CircRNAs have a closed circular structure, lack of the 5ʹ and 3ʹ termini characteristic of traditional linear RNAs, which gives them unique advantages regarding stability and function [7]. CircRNAs perform various biological functions in cells, primarily by acting as microRNAs (miRNAs) sponges, regulating RNA-binding protein interactions, and influencing gene expression, and consequently governing critical cellular processes including cell proliferation, differentiation, apoptosis, and endocrine activities [8]. With the extensive application and advancement of high-throughput sequencing technologies across diverse biological research domains, it has been discovered that circRNAs are not only essential for muscle [9–11], intestine [12], and nerves [13], but are also closely related to the production, differentiation, and maturation of biological germ cells. The expression of numerous circRNAs is closely associated with follicular growth, development, and hormone synthesis [14–17]. CircRNAs are involved in follicular development through various mechanisms, including regulating the dynamic balance of follicle growth by competitively binding to miRNAs, interacting with RNA-binding proteins, modulating gene splicing and translation processes, and regulating follicular endocrine function. For instance, Xie et al. [18] detected circRNAs in the follicles of Meishan and Duroc pigs at the M2 period (5.0–6.9 mm in diameter) using high-throughput sequencing technology. They through GO and KEGG pathway enrichment analyses of host genes linked to differentially expressed circRNAs, they discovered that circRNAs were associated with metabolic regulation, enzyme activity, endocytosis, steroid hormone biosynthesis, cell cycle control, cell adhesion, and homologous recombination. Furthermore, important signaling pathways, including TGF-β, p53, insulin, oocyte meiosis, and the PI3K-Akt pathway, were significantly enriched [18]. These results highlight the widespread presence and functional importance of circRNAs in promoting follicular development. These molecular-level regulations may affect the normal functioning of the reproductive system at various stages of follicular development.

Although the role of circRNAs in follicle development has gradually gained attention, their specific mechanisms and functions have not yet been fully elucidated. Currently, experimental verification regarding the molecular regulation of follicle development and function by circRNAs remains limited. Moreover, while most related studies show that circRNAs possess a certain degree of evolutionary conservation across different mammalian species, this field is still in the exploratory phase. Therefore, this study focuses on analyzing the molecular mechanisms by which circRNAs influence follicle development through the regulation of oocytes, GCs, and endocrine functions. We comprehensively discuss the formation, characteristics, and diverse biological roles of circRNAs, aiming to provide new insights into the regulation of mammalian follicle development by circRNAs and to offer consistent evidence for cross-species research. This paper synthesizes the functions of circRNAs in follicular development, providing critical clues for exploring the molecular basis of follicular developmental abnormalities, supplying researchers with the latest technical tools and research ideas, and promoting the application of new technologies in this field.

## Overview of circRNAs

### The discovery of circRNA and its study in mammalian reproduction

Covalently closed circRNAs, which are pathogenic in certain plants, were first reported as viroids in 1976 [19]. Subsequently, with advancements in high-throughput RNA sequencing and bioinformatic technologies, more studies have identified or synthesized circRNAs across a range of species, including viruses [20], prokaryotes [21], unicellular eukaryotes [21, 22], and mammals [23]. In 2014, researchers first identified the expression patterns of circRNAs in Drosophila ovarian tissues [24]. Since then, circRNAs have been identified in mammalian ovaries. In 2018, Fu et al. [25] first constructed the expression profiles of circRNAs for BMP15 and GDF9 in bovine cumulus cells. In the same year, Tao et al. [26] conducted in-depth sequencing and analysis of circRNAs in pre-ovulatory ovarian follicles of goats. In 2019, Cao et al. [27] first demonstrated that circRNAs are abundantly and dynamically expressed in a development stage-specific manner in pig cumulus cells and oocytes.

### Formation and classification of circRNAs

**Fig. 1:**
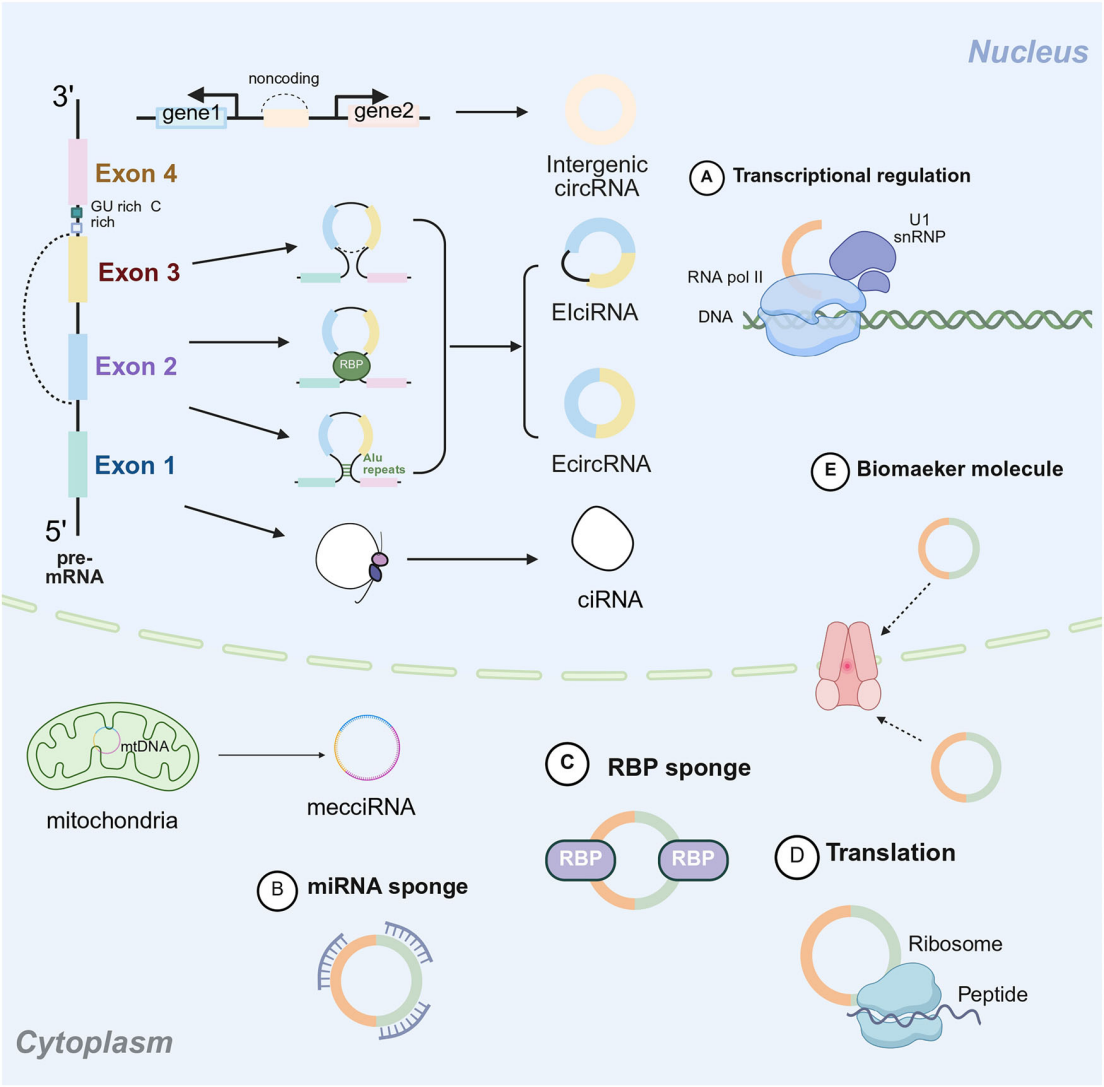
Formation of circRNAs and their biological functions. This figure illustrates the production and functional mechanisms of circRNAs in the nucleus and cytoplasm. Within the nucleus, RNA polymerase II (RNA pol II) uses DNA as a template to transcribe pre-mRNA. These pre-mRNAs undergo processes such as intron pairing (mediated by GU-rich and C-rich elements) or exon skipping to form several types of circRNAs, including exon circRNAs (EcircRNAs), exon-intron circRNAs (EciRNAs), and intergenic circRNAs. When circRNAs are transported from the nucleus to the cytoplasm, they exhibit additional functions. Among these is the mitochondrial circRNA, which is produced directly in the mitochondria. These circRNAs are involved in a variety of cellular functions: **A** they can participate in transcriptional regulation, for example, by interacting with U1 snRNPs; In the cytoplasm, circRNAs play various roles, including: **B** acting as miRNA sponges to adsorb miRNAs, **C** serving as RNA-binding protein (RBP) sponges to regulate gene expression, and **D** functioning as translation templates for ribosome recognition and synthesis of polypeptides, thereby participating in protein synthesis. **E** they can also serve as biomarkers. Notably, circRNAs from different sources intersect in their biological functions, suggesting that they may interact and cooperate within the cell’s regulatory network. Created with Biorender.com.

Based on current research findings, we systematically reviewed and categorized circRNA biogenesis, summarizing the formation of circRNAs. We began by examining the generation of common circRNAs during post-transcriptional modifications through splicing mechanisms. According to different cyclization methods, circRNAs are mainly formed in five ways. Exon-derived circRNAs are the most common type of circRNAs, produced through precursor mRNA (pre-mRNA) backsplicing, and are mainly derived from exons [28]. Intron-derived circRNAs are generated through intronic reverse splicing mechanisms and predominantly localize within the nuclear compartment [29]. Exon-intron circRNAs are formed by exons and introns, and all introns are spliced during the formation of exon-intron circRNAs [30]. Intergenic circRNAs originate from genomic regions outside the coding locus and are flanked by the same GU-AG dinucleotides [31, 32]. MecciRNA is a circRNA encoded by the mitochondrial genome, Similar to nuclear-encoded circRNA, mecciRNA has its 3’ and 5’ ends connected by covalent bonds to form a circular structure [33, 34]. These classifications reflect the diversity and complexity of circRNAs involved in gene expression regulation and various biological functions through different generation mechanisms **(**Fig. 1**)**.

### The characteristics of circRNAs

CircRNAs are characterized mainly by their closed-loop structure, which makes it more stable than linear RNA, resulting in a half-life in cells that is much longer than that of linear RNA, typically exceeding 48 h [35], while the average half-life of mRNA is about 10 h [36], and circRNA is not easily degraded by most exonucleases [37]. Additionally, the formation of circRNAs relies on a special splicing mechanism known as backsplicing, where an intron from a pre-mRNA is excised and two or more exons are linked together to create a loop [38]. CircRNAs display tissue- and developmental stage-specific expression patterns, meaning their expression varies among different tissues and developmental stages [7, 24]. The sequences of circRNAs may not be conserved across species, but their backspacing sites are conserved in some cases [35, 39]. CircRNAs participate in numerous biological processes, such as functioning as sponges for miRNAs, regulating gene expression, participating in protein translation, and influencing RNA stability and splicing [40, 41]. Diverse factors regulate the expression of circRNAs, and these factors serve as important pathways through which circRNAs contribute to the development of various diseases [42, 43]. Additionally, circRNAs are capable of interacting with other RNA molecules and proteins, thereby forming complex gene regulatory networks [44]. These features make circRNAs important molecules for studying gene expression regulation, disease mechanisms, and their promising applications in diagnosis and treatment.

### The biological functions of circRNAs

By acting as sponges for miRNAs, circRNAs can indirectly regulate the expression of downstream target genes [45, 46]. Certain circRNAs possess one or more binding sites for RNA-binding proteins, which can act as sponges for protein molecules. Among them, *circURI1* directly interacts with heterogeneous ribonucleoprotein M (hnRNPM) to regulate the selective splicing of genes involved in cell migration processes, thus inhibiting gastric cancer metastasis [42]. CircRNAs act as transcriptional regulators that activate parental genes, thereby enhancing their expression [47, 48]. Some circRNAs can act as translation templates encoding functional small peptides that regulate biological processes, such as heat-shock stress, tumor cell migration and invasion, and muscle damage [49–51]. CircRNAs also have a significant role in the antiviral immune response. In addition, tumor cells produce crypto peptides through circRNAs, which can be presented to T cells by Major Histocompatibility Complex (MHC) class I molecules to elicit specific immune responses [52], and circRNAs can also be involved in the onset and progression of autoimmune diseases by modulating immune responses and inflammatory processes [53]. CircRNAs can be transmitted between cells through extracellular vesicles such as exosomes, participating in intercellular communication and information exchange [54]. CircRNAs can also participate in epigenetic regulation. The *circRNA FECR1*, originating from the *FLI1* gene, can interact with the promoter of *FLI1* and regulate gene expression by recruiting the demethylation enzyme TET1, leading to demethylation in the CpG island region of the *FLI1* gene [55]. Recently, biomarker-related studies have indicated that circRNAs, with their highly stable internal structures [24], high conservation [35], highly specific expression patterns [56], and broad-spectrum expression [57], can also be used as markers for basic diagnoses, such as abnormal expression in cancer [58], cardiovascular diseases [59] and neurodegenerative diseases [60], with diagnostic and prognostic potential. At the same time, circRNAs are capable of maintaining cell homeostasis and regulating the cell cycle and apoptosis, such as *circCCDC66* stimulates the proliferation of colon cancer cells [61], *circTLK1* aggravates apoptosis in kidney injury [62], and secondly, the expression of certain circRNAs can affect cell adaptability when hypoxia or DNA damage occurs [63]. These functions suggest that circRNAs are crucial in various cellular biological processes within the cell, and their possible involvement in disease initiation and progression has been extensively investigated. The main functions of circRNAs are shown in Fig. 1.

## CircRNAs regulate mammalian follicular development

The maturation of oocytes in the ovaries depends on the multi-level transformation of the follicular structure and the precise synergy of the molecular network. During the female embryonic stage, a reserve of primordial follicles is formed, each of which is composed of meiosis-blocked oocytes and a single layer of flat granulosa cells, and enters a metabolically resting state in the neonatal period until reproductive endocrine signals activate their development [42]. After puberty, specific primordial follicles are activated under the regulation of cyclic hormones: granulosa cell morphology changes from flat to cubic and proliferates, marking primary follicle formation. At this stage, granulosa cells synthesize steroidal precursor material, which lays the foundation for estrogen production. As the expression of the follicle-stimulating hormone (FSH) receptor is upregulated, the follicle enters the secondary stage, and granulosa cells reorganize to form hormone-containing cystic cavities. The follicular membrane tissue differentiates into an outer vascularized membrane cell layer and an inner steroid synthesis structure [64]. The dominant follicle reaches maturity with a diameter of 20 mm, and its cyst cavity is filled with follicular fluid, which induces endometrial thickening through positive estrogen feedback. The mid-menstrual luteinizing hormone (LH) peak triggers the activation of follicle wall proteolytic enzymes, prompting follicle rupture to release oocytes [65]. After ovulation, the residual structure luteinizes and secretes progesterone to maintain the endometrial secretory phase. This process is modulated by the hypothalamic-pituitary-ovarian axis: FSH drives granulosa cell proliferation and estrogen production, while LH primarily controls ovulation and luteal formation, reflecting the spatiotemporal precision of the multi-level regulatory network. CircRNAs have emerged as a focal point in reproductive research owing to their robust stability and tissue specificity. Research indicates that circRNAs exhibit distinct expression patterns during primitive follicle activation, granulosa cell function maintenance, and follicular atresia, and may regulate the developmental process through PI3K/Akt, Wnt/β-catenin, and other pathways [66]. In addition, the aberrant expression of circRNAs is strongly associated with polycystic ovary syndrome (PCOS) and premature ovarian insufficiency (POI), suggesting a potential role in the pathological mechanism [67, 68]. However, the spatiotemporal expression profile, functional mechanism, and relationship between ovarian circRNAs and hormone synergistic regulation still need to be analyzed. The following provides a novel viewpoint for further uncovering the molecular mechanisms of follicle development, highlighting the regulatory network and clinical importance of circRNAs in oocytes, granulosa cells, and hormone and ovarian-related diseases.

### CircRNAs regulate mammalian follicular development through oocytes

**Fig. 2:**
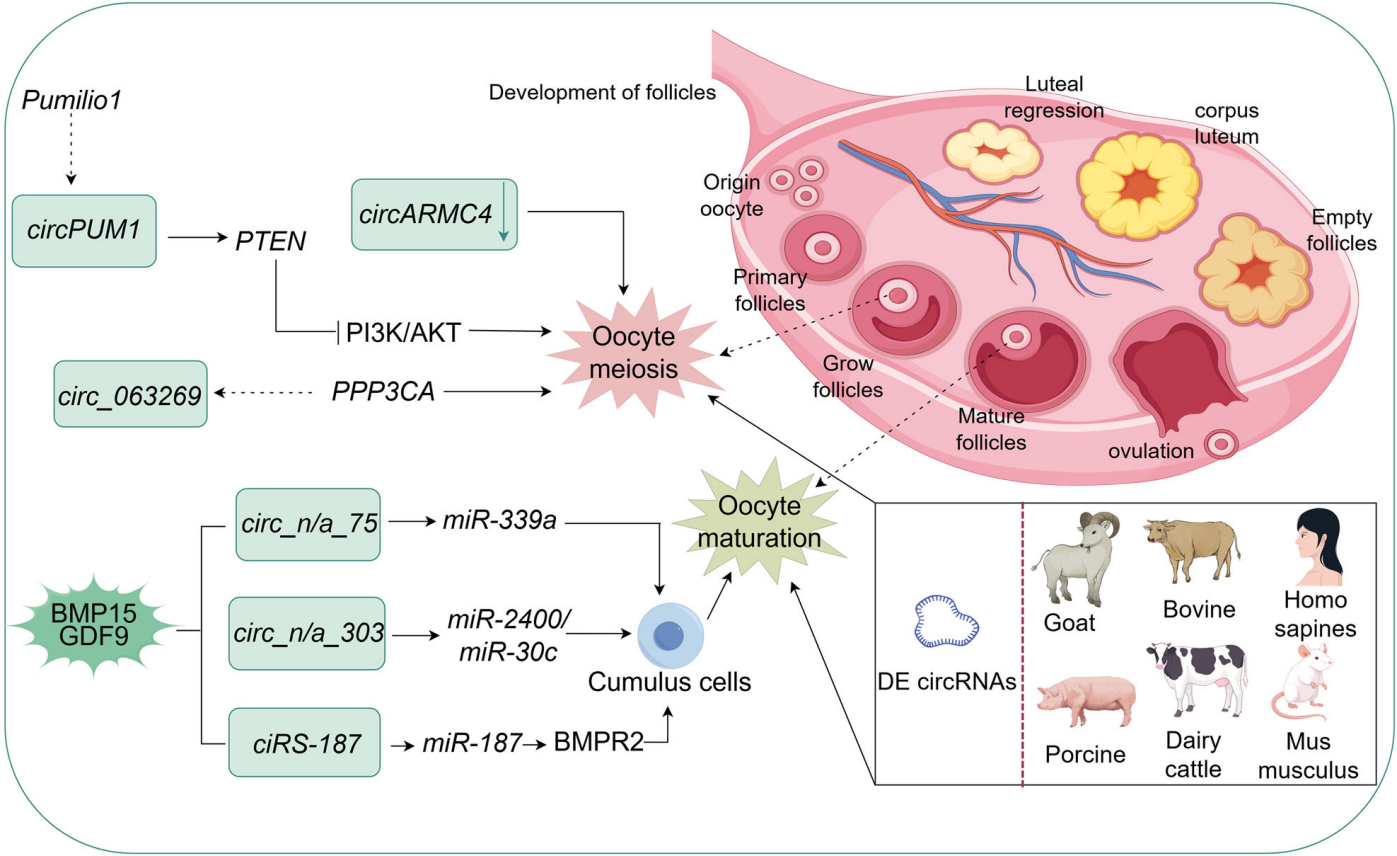
Role of circRNAs in oocyte development. This schematic illustrates the possible connections and expression correlations between circRNAs and miRNAs during oocyte meiosis and maturation. Each box represents a circRNAs, the gene is indicated by italics, the dashed arrow indicates that the circRNAs is derived from the gene, the arrow indicates that it is involved in and regulates the process, the straight arrow indicates inhibition of the process, the parentheses in the upper left panel indicate the specific circRNAs in cumulus cells treated with BMP15 and GDF9, and the green down arrow represents knockdown circRNAs. Among them, BMP15, bone morphogenetic protein 15; GDF9, growth differentiation factor 9, BMPR2 bone morphogenetic protein receptor 2, PTEN phosphodiesterase, and tensor protein homologs, PIK3/AKT indicates the pathway. Created with Figdraw.com.

Oocytes are derived from oogonial differentiation and development via meiosis during oogenesis. Depending on the stage of development, oocytes can be divided into primary, secondary, and mature oocytes, which correspond to the differentiation of oogonia into DNA replication division, the first meiotic division and the products resulting from the second meiotic division, respectively. Recently, circRNAs have demonstrated significant roles in the maturation of mammalian oocytes, such as pigs [27], goats [69, 70], cows, and humans. The specific expression of circRNAs during oocyte maturation not only affects follicular development, but also serves a vital regulatory function in early embryonic development. The knockdown of *circARMC4* in porcine oocytes significantly impairs chromosome alignment during meiotic maturation, thereby disrupting normal embryonic development [27]. In addition, numerous differentially expressed circRNAs were identified in follicles from different developmental stages of black sheep in Yunshang and Dazu, and were notably abundant in pathways associated with oocyte maturation, steroidogenesis, and oocyte meiosis. Among these, *circ-005179* was closely associated with meiotic arrest and resumption [69, 70]. Meiotic resumption refers to the cell cycle in which oocytes are arrested in the first meiotic prophase after birth until meiosis is restarted by hormonal stimulation on the eve of ovulation [71, 72]. In addition, circRNAs can serve as miRNA sponges, thereby regulating various signaling pathways related to reproduction, such as Gonadotropin-Releasing Hormone (GnRH) signaling, oocyte meiosis, and progesterone-mediated oocyte maturation. Mao et al. [73] analyzed RNA expression in the hypothalamus of high- and low-fertility black goats using high-throughput sequencing, and found that 205 differentially expressed circRNAs host genes displayed significant clustering in *PPP3CA* and other reproduction-related GO terms. Furthermore, circRNAs-mRNAs regulatory network analysis indicated that the differentially expressed *circ_063269* originates from *PPP3CA*, which is critically involved in the regulation of estrogen signaling and oocyte meiosis. In addition, Fu et al. [25] found that specific circRNAs regulate the proliferation and differentiation of bovine cumulus cells (CCs) by targeting miRNAs and constructing circRNA expression profiles of bone morphogenetic protein 15 (BMP15) and growth differentiation factor 9 (GDF9) in bovine CCs. Of these, *circ_n/a_75* targets *miR-339a*, and *circ_n/a_303* targets *miR-2400* and *miR-30c*. Another study found that *ciRS-187* promoted the upregulated expression of bone morphogenetic protein receptor 2 (BMPR2) in BMP15 and GDF9-treated bovine-ovine thalamus cells by targeting *miR-187*. Secreted by oocytes, BMP15 and GDF9 belong to the transforming growth factor β (TGFβ) superfamily [74], whereas BMPR2 is a TGFβ type II receptor expressed on follicular cells. GDF9 and BMP15 regulate the downstream metabolic responses by binding to BMPR2 and other type I receptors (Type I receptors refer to the first class of receptors for TGFβ superfamily members. Common Type I receptors include Bone Morphogenetic Protein Receptor Type 1 A (BMPR1A), Bone Morphogenetic Protein Receptor Type 1B (BMPR1B), and Activin a Receptor Type 1 (ACVR1)) [75, 76]. In addition, they bind to receptors on the oocyte, triggering a cascade of downstream gene expression, which in turn affects oocyte proliferation and apoptosis, thereby regulating follicular development and oocyte maturation [77]. Caponnetto et al. [78] found that *circPUM1*, derived from the *Pumilio 1* gene, regulates the expression of phosphodiesterase and tensor protein homolog (*PTEN*) in human follicular cells. *PTEN* is a key molecule in maintaining the quiescent state of the primordial follicular pool and regulating primordial follicle survival and activation. It achieves these functions through suppression of the PI3K/AKT signaling pathway and modulation of cycling follicle recruitment. This process ultimately leads to GC ovulation and stimulates the resumption of oocyte meiosis [79, 80]. In summary, circRNAs participate in the regulation of oocyte maturation and follicle development through various molecular mechanisms. However, there are still knowledge gaps regarding their specific functions in the mammalian reproductive system, necessitating more in-depth experimental validation. As shown in Fig. 2, based on existing research and circRNAs-miRNAs predictive analysis, a potential regulatory network model of circRNA in oocytes has been proposed, but this hypothesis still requires confirmation through subsequent functional experiments.

### CircRNAs regulate mammalian follicular development through GCs

**Fig. 3:**
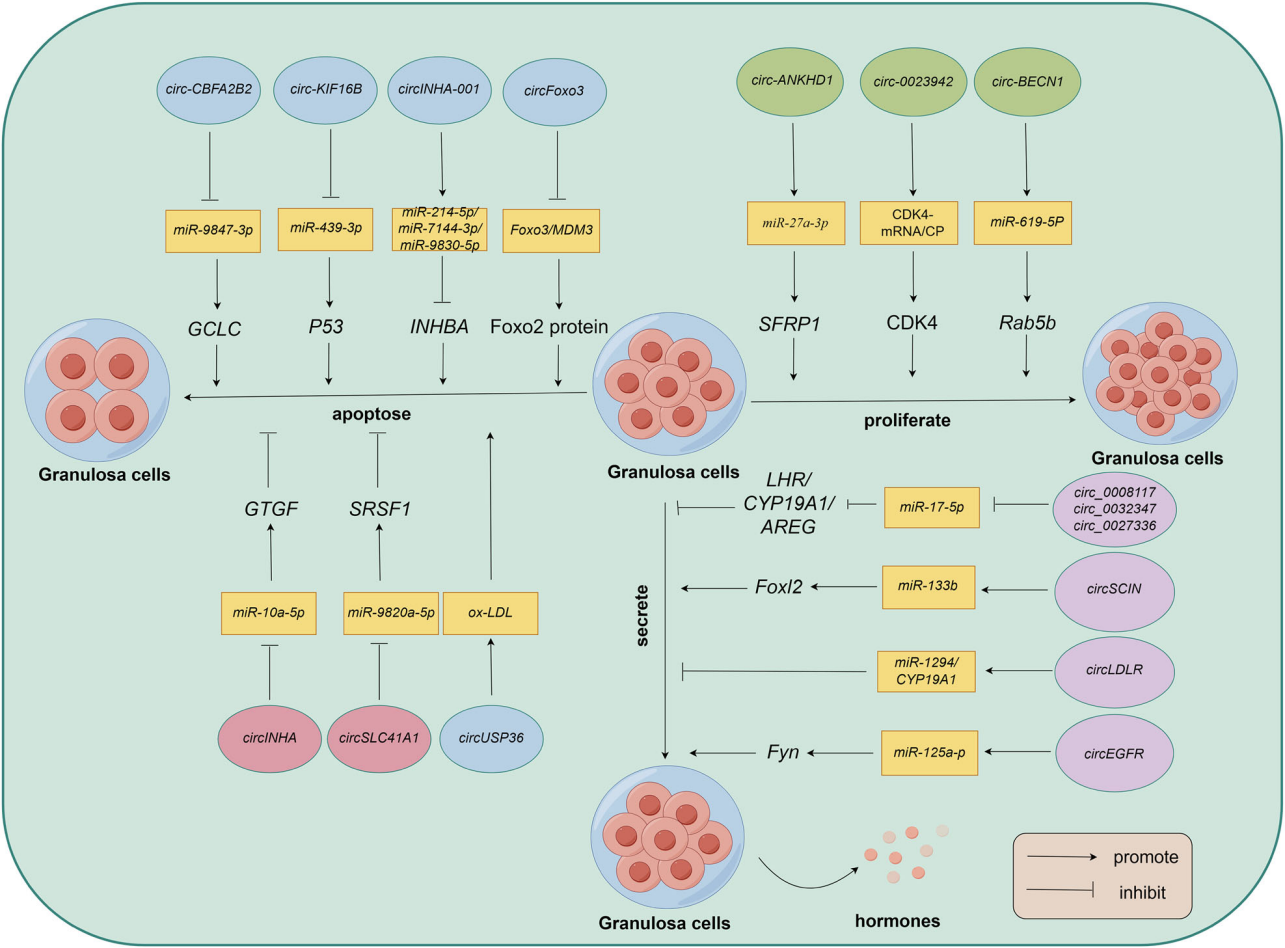
The role of circRNAs in granulosa cell development. This schematic diagram illustrates the involvement of circRNAs in the processes of granulosa cell proliferation, differentiation, apoptosis, and endocrine function. The diagram delineates the specific circRNAs that modulate these cellular activities, with each circRNAs depicted as a circle. Created with Figdraw.com.

GCs are an important component of the ovarian follicle that closely surrounds the oocyte and provides support and protection. During follicular development, GCs start to differentiate from a single layer of flat cells at the primordial follicular stage, progressively develop into a single layer of cuboidal GCs, and then proliferate to form a multilayered structure that eventually develops into a luminal follicle containing GCs of the oocyte mound and GCs of the wall. GCs play a central role in follicular development, ovulation, and hormone secretion, and are responsible for the secretion of steroid hormones like progesterone and estrogen, as well as for regulating oocyte growth and maturation through cell-to-cell signaling and metabolic support. The equilibrium of GC proliferation and apoptosis is fundamental to follicular development and atresia. Alterations in circRNAs expression are regulated by miRNA sponges. Researches have already covered many circRNAs and even determined their roles in GC proliferation as well as apoptosis. Such as one study observed notable expression differences exist in specific circRNAs like *circ_0015292* and *circ-0001651* in the follicles of Meishan pigs compared with those in Duroc pigs. Further studies revealed that *circ-0015292* (downregulated) was able to bind *miR-181-a*, which is associated with follicular development, and *circ-0001651* (upregulated) contained 18 potential binding sites associated with eight different miRNAs that regulate follicular granule cell development [18]. Similar findings were reported in a study on long white pigs, where *circ-52,011*, *circ-30,716*, *circ-58,954*, and *circ-79,388* were found to have high-density binding sites for *miR-339-3p*, *miR-214-3p*, and *miR-708-5p* [81]. Notably, *miR-214-3p* serves as a cardinal regulator in both the lncRNAs-miRNAs-mRNAs and circRNAs-miRNAs-mRNAs networks, similarly influencing follicular development in both mouse and porcine ovaries [79, 82]. These results highlight the regulatory role of circRNAs in ovarian GCs and their potential impact on reproductive processes. Fig. 3 illustrates the potential action network of circRNAs in granulosa cells, with this schematic diagram proposing a hypothetical model based on existing data, the specific mechanisms of which still require experimental validation.

### CircRNAs regulate mammalian follicular development by affecting GC proliferation and differentiation

The proliferation and differentiation functions of GCs play a pivotal role in follicle maturation, atresia and the development and maturation of oocyte [83]. CircRNAs regulate GC proliferation and differentiation, mainly by acting on miRNAs and target genes involved in the cell cycle and proliferation. One study revealed that many circRNAs exhibit distinguishing expression in atretic follicular GCs in contrast with healthy follicular GCs. Among them, *circ-ANKHD1* expression was 10-fold lower in atretic follicles in sows than that in healthy follicles. Further studies have shown that *circ-ANKHD1* promotes GC proliferation while simultaneously decreasing apoptosis by acting on the *miR-27a-3p*/SFRP1 signaling pathway [15]. In addition, in healthy buffalo follicles, *circTEC* exhibited high level of expression and acted as a natural sponge for *miR-144-5p*. This interaction regulated the expression of the target gene *FZD3* in antral follicles, stimulating buffalo granulosa cells to proliferate as well as promoting the synthesis of steroid hormones and inhibited their apoptosis [84]. Similarly, *circ_0023942* expression has a strong relation to GC proliferation and apoptosis. By transiently transfecting the *circ_0023942* overexpression vector into human ovarian granulocyte cell lines, *KGN* and *COV434*, *circ_0023942* was discovered with a suppression to the expression of cell cycle-dependent protein kinase 4 (CDK4) at both the mRNA and protein levels [85]. CDK4 acts a key factor in advancing cells from the G1 to S phase and induces proliferation of cells [86]. In addition, the differential *circCFAP20DCs* in goat follicles enhanced cell proliferation by promoting GC transition from the G1 to S phase, demonstrating substantial biological effects [66]. Many circRNAs were enriched in ovarian steroidogenesis, the GnRH signaling pathway, the oxytocin signaling pathway, the animal autophagy pathway, as well as reproduction, which was further validated by studying goat follicles of different sizes and identifying differentially expressed circRNAs [66]. These studies reveal the important potential of circRNAs in regulating follicular development and reproductive function.

### CircRNAs regulate mammalian follicular development by affecting GC apoptosis

Excessive or disorganized apoptosis of GCs is a sign of impaired oocyte development. This phenomenon disrupts the close association between oocytes and GCs during follicular development, inhibits oocyte growth, and accelerates follicular atresia, thereby affecting ovarian function and decreasing biological reproductive capacity. GC apoptosis, a critical component of follicular growth, development, and maintenance, is a major cause of follicular atresia. Many circRNAs are differentially expressed in porcine atretic follicular GCs compared with those in normal follicular GCs and affect GC apoptosis. One study detected 197 differentially expressed circRNAs in atretic follicles, with 108 upregulated and 89 downregulated, while *circINHA* expression was significantly downregulated in porcine follicular atresia. Further studies showed that *circINHA*, through a competitive endogenous RNA (ceRNA) mechanism directly bound to *miR-10a-5p*, regulates connective tissue growth factor, thereby inhibiting apoptotic GCs and promoting proliferation [87]. Similarly, another study reported that *circSLC41A1* expression was downregulated during porcine follicular atresia and *circSLC41A1* inhibited GC apoptosis by competitively binding to *miR-9820-5p* [88]. Additionally, another study showed that 62 circRNAs exhibited significant expression differences in atretic follicles. KEGG analysis of their host genes revealed apoptosis as a significantly enriched pathway. Among them, *circ-CBFA2T2* and *circKIF16B* regulate the antioxidant gene glutamine-cysteine ligase catalytic subunit (*GCLC*) and apoptotic gene p53 (*TP53*) through the adsorption of *miR-9847-3p* and *miR-493-3p*, respectively, which promote GC apoptosis and follicular atresia, leading to impaired porcine oocytogenesis [89]. In contrast, another study showed that *circINHA-001* was expressed at higher levels in healthy porcine follicles. *CircINHA-001* functions via the adsorption of three miRNAs that share the same target as *INHBA*. These miRNAs are respectively *miR-214-5p*, *miR-7144-3p*, and *miR-9830-5p*. Reduced expression of *circINHA-001* increases free miRNAs levels and inhibits *INHBA* expression, thereby enhancing GC apoptosis via an alteration from activin to inhibin secretion [90]. Furthermore, in human studies, different circRNAs can regulate granulocyte function through different miRNA-mRNA axes. For instance, through regulating the interaction between *miR-619-5p* and *Rab5b*, *circ_BECN1* stimulates the proliferation of ovarian GCs, facilitates the progression of the cell cycle, and diminishes apoptosis [91]. *CircFoxo3* promotes GC apoptosis by modulating FOXO2 protein levels and reducing the interaction between *FOXO3* and *MDM3* [92]. *CircUSP36* is linked to the process of apoptosis and heightened autophagy in GCs triggered by oxidized low-density lipoprotein (ox-LDL) [93]. Finally, some researches have observed that circRNAs expression in GCs is regulated by nutrient-induced GC function. Zhang et al. [94] investigated resveratrol-induced circRNAs expression in porcine GCs and found that these circRNAs are involved in a variety of cellular activities, including oxidative stress, cell metabolism, estrogen receptor (ER) binding, and apoptosis. In addition, *EIF4A3*-mediated exosomes *circLRRC8A* mitigate GC in ovaries of patients with premature ovarian failure aging by modulating the *miR-125a-3p*/*NFE2L1* signaling pathway [95].

### CircRNAs regulate mammalian follicular development by modulating steroid secretion in GCs

GCs express the FSH and ER on their surface. The combination of FSH and the receptor induces aromatase synthesized successfully, which promotes the conversion of androgens to β-estradiol (E2) by GCs. GCs play a vital part in maintaining endocrine balance through secreting β-estradiol [96, 97]. The secretion of β-estradiol not only maintains endocrine homeostasis, but also inhibits GC apoptosis and accelerates follicle maturation through synergistic effects with various hormones and related factors [98]. Recently, circRNAs have important biological functions in GCs. CircRNAs participate in regulating steroid hormone production and act like sponges for miRNAs to competitively bind to them, thereby inducing downstream target genes to express and regulating reproductive functions of both humans and animals. Animal reproductive function. Wang et al. [99] discovered that the expression of *circ_0008117*, *circ_0032347*, *circ_0027336*, and *ZFPM2* was markedly elevated in the ovarian tissues of black and Hetian sheep during the oestrus period, and these circRNAs collectively downregulated *miR-17-5p*, which makes important sense for ovarian function [100]. In addition, *miR-17-5p* upregulation enhances the expression of follicular development marker genes (e.g. *LHR, CYP19A1*, and *AREG*) [101, 102], suggesting that circRNAs are involved in the regulation of follicular development by modulating *miR-17-5p*. Other mechanisms of action for circRNAs have also been validated. *CircEGFR* is highly expressed in adult mouse ovaries and enhances the proliferation of granule cells and the secretion of estradiol by binding to *miR-125a-3p* and regulating the expression of the *Fyn* gene [103]. In addition, it was found that the lack of *circLDLR* in Polycystic Ovary Syndrome reduces estrogen synthesis by affecting the expression of *miR-1294* and *CYP19A1* [16]. Another study has indicated that *circDDX10* regulates in female ovarian granulosa cells related to steroidogenesis and promotes β-estradiol synthesis [17]. Liang et al. [104] found that the differentially expressed *circSCIN* in Meishan pigs stimulates the synthesis of ovarian β-estradiol by adsorbing *miR-133b* and downregulating the expression of *Foxl2* in GCs [105]. In addition, *miR-133b* is closely associated with FSH-induced estrogen secretion. Therefore, we hypothesized that *circSCIN* affects estrogen synthesis in GCs by interacting with *miR-133b*. Pan et al. [14] showed that the target gene of *circRNAs 10:22806071-22812591* is *NR5A2*, whose enhanced expression promotes luteinizing hormone synthesis in porcine GCs [106], suggesting that circRNAs hold extreme importance in steroid synthesis through modulating *NR5A2* expression. Li et al. [107] unfolded that continuous light exposure leads to abnormal expression of circRNAs in the ovaries of rats, which may interfere with follicular development and sex hormone secretion, and affect reproductive function by participating in a variety of biological processes and signaling pathways. In summary, circRNAs regulate steroid hormone secretion in GCs through multiple mechanisms and promote follicular development by means of modulating related genes to express (Fig. 3).

### CircRNAs regulate follicular development through gonadotropins

Gonadotropins promote follicular development until maturation by stimulating the proliferation and differentiation of follicular cells. An important component of gonadotropins is FSH, which plays a key role in follicular development and maturation, interacting with LH to regulate animals’ steroid hormone production and physiological activities [108]. FSH helps follicles reach maturity, whereas once the follicle has matured, LH brings follicle a break and ovum to release, which completes the ovulation process. Gonadotropins are crucial for follicle growth, development, maturation, and ovulation. CircRNAs have already been demonstrated to be induced by gonadotropins and functioning as miRNA “sponges” to regulate the manifestation of key genes which contribute to follicular development. This regulatory mechanism consequently influences folliculogenesis and reproductive function. Specifically, Chakravarthi et al. [109], using miRNA screening analysis, investigated how miRNAs interacting with LH-induced circRNAs are related in function, and furtherly constructed a network for these miRNAs together with their target mRNAs. They ascertained the key combination, *circVcan/has-miR-326*/*FSHR*. *FSHR* is a coreceptor in the FSH signaling cascade and also a decisive part in ovarian follicular development. This suggests that LH-regulated circRNAs function as miRNA sponges that regulate the representation of key genes necessary for ovarian follicular development. Additionally, *circRNAs4464*, derived from *EFNA5*, shows marked differences in expression between small and large follicles; it was upregulated in small follicles and may hinder follicular development by inhibiting the follicular cell response to FSH [110]. Researches in mice have shown that *EFNA5* is vital for gonadotropin activity and ovulation process, and *EFNA5* deficiency leads to reduced fertility [111]. In addition, FSH promotes the expression of *EFNA5* in sheep follicles, further indicating its important role in follicular development in animals [112]. In another study, by analyzing the transcriptome of the sheep hypothalamus, circRNAs associated with *FecB* mutations were characterized, and their expression profiles were identified, highlighting their potential role in the follicular-luteal transition. Functional enrichment analysis revealed that some differentially expressed circRNAs are important for oocyte maturation and hormone signaling pathways. In addition, ceRNA network analysis indicated that *circ_0000523* and *circ_0028984* may affect ovulation by regulating LH synthesis and secretion [113]. These discoveries offer fresh perspectives on how circRNAs regulate follicular development and ovulation (Table 1).

**Table 1:** Role of circRNAs in mammalian follicle development.

CircRNAs	Expressions	Target miRNAs	Function energy of circRNAs	Species	References
*circ0008219*	Up	*miR-34c-5p, miR-483, miR-1468-3p*	Regulation of follicular growth to prevent follicular occlusion leading to granulosa cell apoptosis	Goat	[26]
*circ-CBFA2T2*	Down	*miR-9847-3p/GCLC*	Promoting follicular atresia	Porcine	[89]
*circINHA*	Down	*circINHA/ miR-10a-5p/GTGFaxis*	Resistance to granulosa cell apoptosis, affecting follicular atresia	Porcine	[87]
*circARMC4*	Up	*_*	Regulation of oocyte maturation and early embryo development	Porcine	[27]
*circ*_*-*_*n/a*_*-*_*75 and circ*_*-*_*n/a*_*-*_*303*	Up	*miR-30c/miR-2400 and miR-339a*	Influence on the proliferation and differentiation of oocytes	Bovine	[25]
*circPUM1*	Up	*PTEN*	stimulates oocyte meiosis recovery	Homo sapiens	[78]
*ciRs-187*	Down	*miR-187*	Promotes BMPR2 expression and affects proliferation and apoptosis of cumulus cells	Bovine	[127]
*circ*_*-*_*0001651*	Up	*miRNAs*	Affects granule cell development	Porcine	[18]
*circ*_*-*_*ANKHD1*	Up	*miR-27a-3p/SFRP1*	Promotes proliferation of granulosa cells	Porcine	[15]
*circ*_*-*_*0023942*	Up	*CDK4*	Inhibition of CDK_4_ mRNA and protein expression, thereby inhibiting granulosa cell proliferation	Homo sapiens	[85]
*circ*_*-*_*KIF16B*	Up	*miR-493-3p/p53*	Promotes apoptosis in granulosa cells	Porcine	[89]
*circINHA-001*	Down	*miR-214-5p, miR-7144-3p, miR-9830-5p/INHBA*	Promotes apoptosis in granulosa cells	Porcine	[90]
*circFoxo3*	Up	*Foxo3/MDM3/Foxo2*	Promotes apoptosis in granulosa cells	Mus musculus	[92]
*circUSP36*	Up	*NEDD4L*	Promotes apoptosis and excessive autophagy in granulosa cells	Homo sapiens	[93]
*circSLC41A1*	Down	*miR-9820-5p/SRSF1*	Inhibition of granulocyte apoptosis	Porcine	[88]
*circEGFR*	Down	*miR-125a-3p/Fyn*	Increases E2 secretion and enhances granulocyte proliferation	Homo sapiens	[128]
*circSCIN*	Down	*miR-133b/Foxl2*	Stimulation of estradiol synthesis	Mus musculus	[104]

## The role of CircRNAs in follicular pathology

**Fig. 4:**
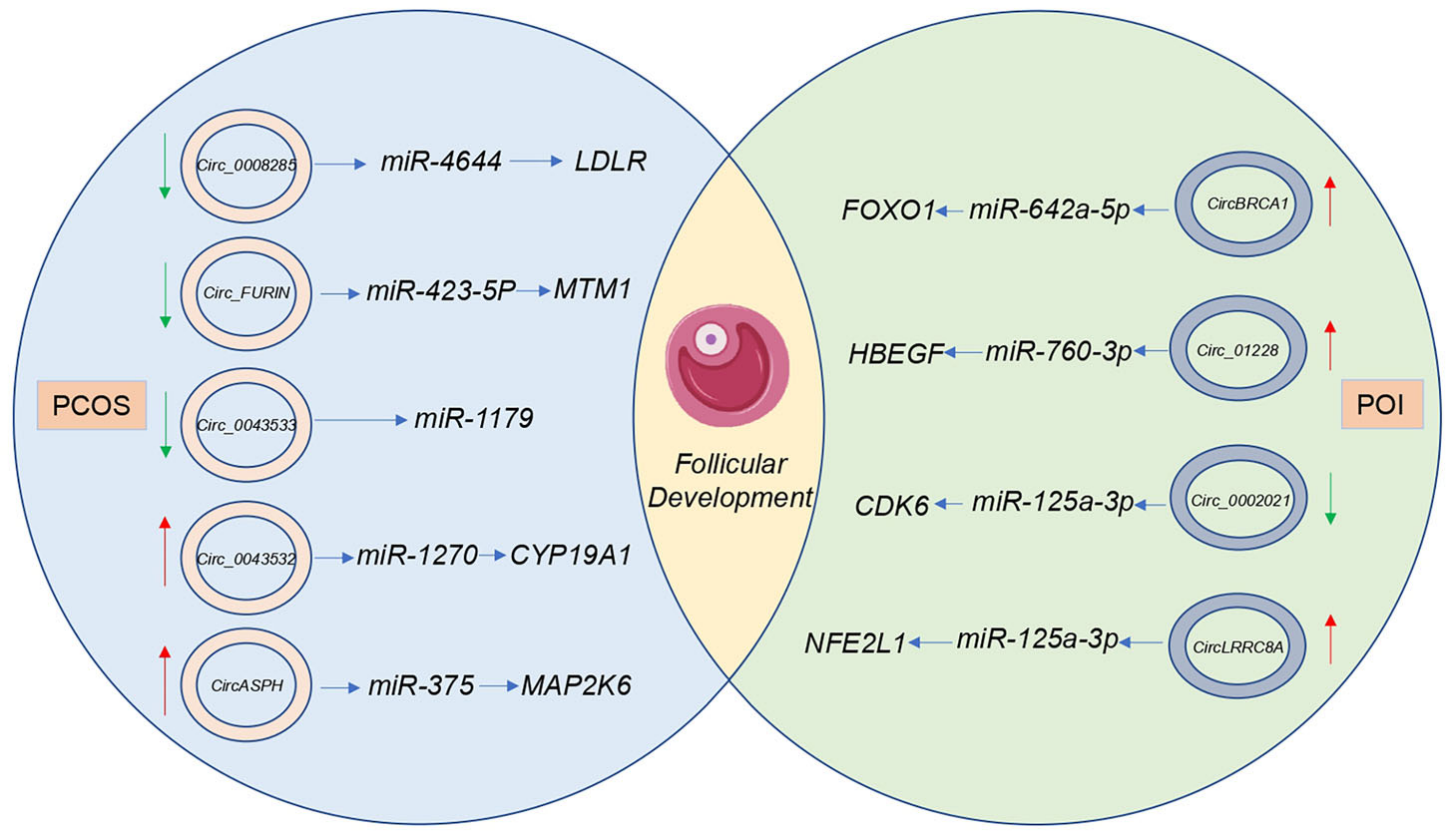
CircRNAs in Follicles of PCOS and OC and Their Functions. This figure delineates the role of various circRNAs in the regulation of follicular dynamics within the context of polycystic ovary syndrome and premature ovarian insufficiency. The graphical representation employs red arrows to denote upregulation and green arrows to signify downregulation of circRNAs expression levels. Each circRNAs is depicted as a circle within the illustration. Created with Biorender.com.

Abnormal follicular growth and development can not only directly impair ovarian function but also lead to a range of reproductive endocrine disorders and metabolic dysfunctions, exerting broad impacts on female health. Among these, the most representative conditions are PCOS and POI. PCOS and POI are two prevalent female reproductive endocrine disorders that, despite their distinct pathological mechanisms, significantly impair patients’ life quality, fertility, and healthy stability. PCOS is primarily characterized by ovarian polycystic changes, hyperandrogenism, and insulin resistance resulting from abnormal follicular development, often leading to menstrual irregularities, infertility, and metabolic syndrome. In contrast, POI is caused by premature follicular pool depletion or impaired follicular maturation, leading ovarian function into a decay before the normal reproductive age. This condition is marked by hypoestrogenism, elevated gonadotropin levels, and menstrual cycle abnormalities, alongside higher risk into osteoporosis and cardiovascular diseases. Both disorders are linked to follicular developmental abnormalities, contributing to endocrine dysregulation, metabolic dysfunction, and mental health issues. However, PCOS is more closely associated with systemic metabolic-reproductive disturbances due to follicular dysregulation, whereas POI is defined by ovarian failure resulting from follicular depletion. Early diagnosis and tailored therapeutic interventions are crucial for improving the prognosis of these conditions (Fig. 4).

In a study on the molecular mechanism of PCOS, exosomes *circ_0008285* in follicular fluid have been found to regulate lipid metabolism by targeting the *miR-4644/LDLR* signaling axis [114]. This finding provides a new molecular perspective for understanding abnormal lipid metabolism in patients with PCOS. Combined with the mechanism of miRNA adsorption by circRNAs, *circ_0043533* was found to function as a molecular sponge for *miR-1179*, mitigating its inhibitory effect on ovarian granulosa cell proliferation and apoptosis through adsorption of *miR-1179*, which may affect follicular development and maturation [115]. Similarly, related studies indicated that *circ_FURIN* reduced the expression level of myosin 1 (*MTM1*), a protein associated with muscle metabolism, by interacting with *miR-423-5p*, and that the downregulation of its expression may be connected to metabolic abnormalities in PCOS patients [116]. The knockdown of *circ_FURIN* alleviates testosterone-induced human ovarian GC disease, suggesting that *circ_FURIN* may be a crucial role in the pathological progression of PCOS [116]. In addition, *circ_0043532*, a ceRNA for *miR-1270*, upregulated the expression of *CYP19A1*, thereby promoting aberrant steroid production in the GCs of patients with PCOS [117]. *CYP19A1*, a key aromatase, takes a central role in steroid synthesis. The regulatory effect of *circ_0043532* to *CYP19A1* may directly affect the endocrine status of patients. This indicates that circRNAs are pivotal in PCOS. In summary, it is demonstrated that circRNAs is indeed significant roles in PCOS pathogenesis through diverse molecular mechanisms, giving promising perspectives to the development of new diagnostic biomarkers and therapeutic interventions.

CircRNAs expression was significantly associated with the occurrence of POI. Given the differential expression of circRNAs in tissues of POI and normal ovarian patients, circRNAs can be used as predictive biomarkers and therapeutic targets. In premature ovarian failure, the abnormal expression of some circRNAs affects the regulation of miRNAs on genes associated with ovarian granulosa cell function, resulting granulosa cells into unbalanced proliferation and apoptosis, which impairs ovarian function in turn. CircRNAs, by interacting with miRNAs and mRNAs, take a crucial role in the progression of POIs and other ovarian diseases, and provide a target for POI therapy. Ge et al. [118] showed that *hsa_circ_0002021*, derived from umbilical cord mesenchymal stem cell exosomes, is effective to improve granulosa cell function in POI. Mechanistically, *circ_0002021* functions as ceRNA for *miR-125a-5p*, alleviating granulosa cell senescence by modulating the manifestation of cyclin-dependent kinase 6 (CDK6), thereby restoring ovarian function in POI models. Secondly, the interaction between N⁶-methyladenosine (m⁶A) modification and circRNA provides new insights into ovarian function. One study had found that *circBRCA1* expression is reduced in the serum and GCs of patients with POI, which is closely related to the decline in ovarian reserve function. Exosomes rich in *circBRCA1* can significantly improve estrous cycle disorders and reproductive hormone levels in POI rats, reduce the number of atretic follicles, and alleviate GCs’ apoptosis and senescence. Mechanistically, the demethylase FTO mediates the m⁶A demethylation of *circBRCA1*, thereby increasing its stability and expression levels. *CircBRCA1* exerts protective effects by adsorbing *miR-642a-5p*, blocking its interaction with *FOXO1* [119]. The function of circRNA can be regulated by m⁶A modification, affecting its biogenesis, stability, and translational potential, m⁶A modification can also drive the initiation of circRNA translation [120, 121]. Meanwhile, m⁶A modification plays a crucial role in folliculogenesis and oocyte maturation, influencing oocyte development and function by regulating mRNA stability, translation efficiency, and maternal-zygotic transition [122–126]. Thus, m⁶A modification can modulate follicle maturation and oocyte development by impacting the biosynthesis, stability, and functions of circRNAs. Currently, research on the relationship between m⁶A modification and circRNA in regulating follicle development is still limited. Based on existing studies, utilizing the interaction of m⁶A modification with circRNA as a research direction for fundamental studies and clinical applications in follicle development holds significant research value.

These findings not only offer further insights into the molecular pathology of PCOS and POI but also accentuate the potential for developing novel diagnostic markers and therapeutic targets. Table 2 and Fig. 4 summarize recently published high-quality literature that underscores the possibility that circRNAs address PCOS and POI as therapeutic targets. CircRNAs show significant promise in this context, with unique capabilities to improve ovarian dysfunction and metabolic dysregulation through their regulatory roles in miRNA interactions, m^6^A modification, signaling pathways, and epigenetic modifications. As research and technology continue to advance, circRNAs are emerging as pivotal tools in the precision therapy of PCOS and POI.

**Table 2:** Molecular mechanisms by which circRNAs regulate ovarian disease.

CircRNAs	Expressions	Target miRNAs	Parental genes	Biological roles	Types of Diseases	References
*circ_0008285*	Down	*miR-4644*	*LDLR*	Promotes cholesterol metabolism in ovarian granulosa cells	PCOS	[114]
*circ_0043533*	Down	*miR-1179*	*_*	decreased the silence on cell proliferation and apoptosis in ovary-related cells	PCOS	[115]
*circ_FURIN*	Down	*miR-423-5p*	*MTM1*	inhibiting granulosa cell proliferation and promoting cell apoptosis	PCOS	[116]
*circ_0043532*	Up	*miR-1270*	*CYP19A1*	Promotes the secretion of estradiol in granulosa cells	PCOS	[117]
*circASPH*	up	*miR-375*	*MAP2K6*	Inhibits KGN cell proliferation and enhances KGN cell apoptosis	PCOS	[129]
*circBRCA1*	Up	*miR-642a-5p*	*FOXO1*	resisted oxidative stress injuries in GCs and protected ovarian function in rats with POI	POI	[130]
*circ_0002021*	Up	*miR-125a-5p*	*CDK6*	Ameliorates in vitro GC senescence by modulating oxidative stress and cellular senescence markers	POI	[118]
*circLRRC8A*	Down	*miR-125a-3p*	*NFE2L1*	Promotes granulosa cell senescence	POI	[95]
*circRNA_012284*	Up	*miR-760-3p*	*HBEGF*	Reduces oxidative stress and improves POI in ovarian granulosa cells	POI	[131]

## Summary and outlook

### Summary

**Fig. 5:**
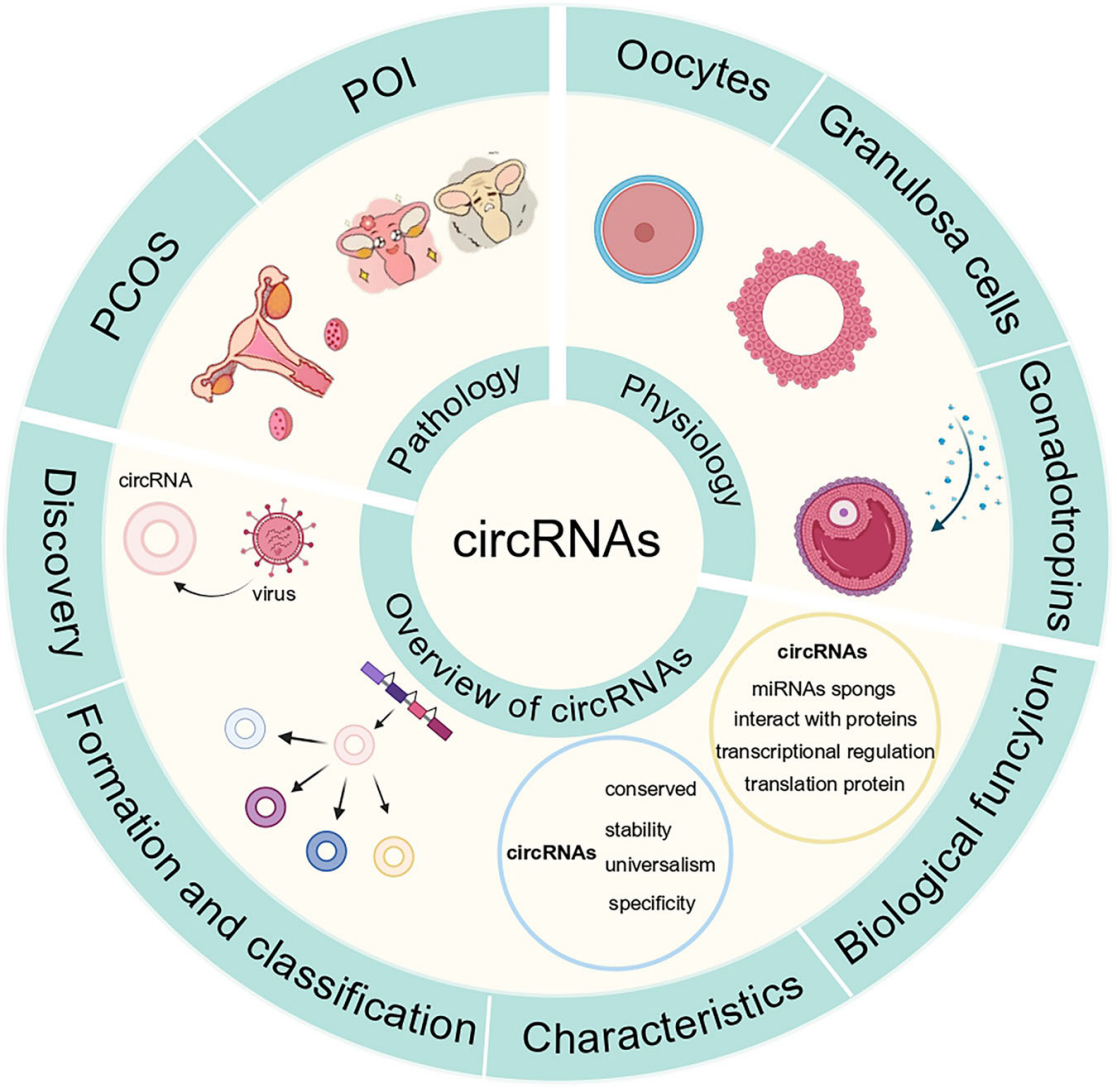
CircRNAs are involved in mammalian follicular development and pathological regulation through their biological functions. Created with Biorender.com.

This study provides an in-depth review about the roles of circRNAs in mammalian follicular development. It summarizes the formation mechanisms, characteristics, and functions of circRNAs, particularly in ovarian GCs and oocytes. Researches indicate that circRNAs take part in cell proliferation, differentiation, apoptosis, and maturation via operating as miRNA sponges, regulating RNA-binding proteins, together with influencing gene expression. Additionally, the study covers the role played by circRNAs in pathological conditions like PCOS and POI, highlighting their potential applications in reproductive medicine and animal husbandry. Furthermore, novel insights into the molecular mechanisms underlying follicular development, and potential directions and strategies for future research to enhance reproductive health and efficiency, were outlined (Fig. 5).

### Outlook

Although we gained an understanding of what role circRNAs play in follicular development, further investigation is still required for the research to their special biological functions. Focus of the future effort is expected on identifying target genes of circRNAs and their regulatory networks, and validating the molecular regulatory mechanisms of circRNAs in follicular development and function, in order to elucidate their specific roles in follicular development. The effect of circRNAs acting as biomarkers or therapeutic targets warrants further investigation. Researchers should deeply dig out the mechanisms of circRNAs in ovarian diseases (e.g. PCOS) so that to give novel ideas in dealing with early diagnosis and treatment. Additionally, with the development of genomics and transcriptomics technologies, the diversity of circRNAs and their functions at the cellular level can be analyzed in depth using advanced technological skills of single-cell RNA sequencing, etc. This will help to reveal their multiple regulatory roles in complex biological processes.
